# Knowledge, attitude and perceptions of medical students towards mental health in a university in Uganda

**DOI:** 10.1186/s12909-022-03774-0

**Published:** 2022-10-20

**Authors:** Raymond Bernard Kihumuro, Mark Mohan Kaggwa, Timothy Mwanje Kintu, Rachael Mukisa Nakandi, David Richard Muwanga, David Jolly Muganzi, Pius Atwau, Innocent Ayesiga, Josephine Nambi Najjuma, Scholastic Ashaba

**Affiliations:** 1 Faculty of Medicine, Mbarara University of Science and Technology, Mbarara, Uganda; 2 African Centre for Suicide Prevention and Research, Mbarara, Uganda; 3 Department of Psychiatry and Behavioural Neurosciences, McMaster University, Ontario, Canada

**Keywords:** Knowledge about mental health, Attitude towards mental health, Perception towards mental health, Medical students, Uganda, Knowledge, Attitude, Perception

## Abstract

**Background:**

The prevalence of mental illness among medical students is high. A gap remains on what knowledge should be given to improve the attitudes and perceptions towards mental health. Despite the vast body of literature globally, no study has been conducted in Uganda to assess the levels of knowledge, attitude, and perception among medical students in Uganda.

**Objective:**

To determine the level of knowledge, attitude, and perception and their associated factors among medical students in Uganda.

**Methods:**

A cross-sectional study was done among 259 undergraduate medical students in a public university capturing information on knowledge, attitude, and perception towards mental health. Linear regression analysis was used to determine the factors associated with knowledge, attitude, and perception.

**Results:**

About 77.72% had high knowledge, 49.29% had positive attitudes, and 46.92% had good perceptions of mental health. There was a significant positive relationship between attitude and perceptions towards mental illness. At multilevel analysis, being in year 4 increased the level of knowledge (*β* = 1.50 [95% confidence interval (CI) = 0.46–2.54], *p* = 0.005) while a positive history of mental illness worsened perceptions towards mental illness (*β* = -4.23 [95% CI = −7.44–1.03], p = 0.010).

**Conclusion:**

Medical students have a high level of knowledge about mental illness but the majority had poor attitudes and perceptions of mental illness. Exposure to psychiatry knowledge about mental illness in year four increased students’ knowledge while prior experience with mental illness conditions was associated with poorer perceptions. The information present in this study can be used by policymakers and future researchers to design future studies and interventions to improve knowledge, perceptions, and attitudes especially among students who have a history of mental illness. Improvements in knowledge, attitude, and perception may improve the mental health services for the future patients of these medical students.

**Supplementary Information:**

The online version contains supplementary material available at 10.1186/s12909-022-03774-0.

## Introduction

According to the World Health Organization (WHO), mental health is a state of well-being where every individual realizes his or her own potential, can cope with the normal stresses of life, can work productively and fruitfully, and is able to contribute to her or his community [1]. Mental illness and substance abuse disorders are leading causes of disability in sub-Saharan Africa, with the region having poor mental health services resources [2–5]. A WHO survey of university students across 21 countries found that one-fifth met the criteria for mental disorders in the past year [6]. A finding attributed to university studies being emotionally and intellectually demanding, making students prone to mental health challenges [7–9].

Medical students are more susceptible to developing psychological distress and mental health disorders relative to other students in undergraduate training [10–12]. For example, the prevalence of depression (i.e., 27.2%), anxiety (33.8%), burnout (12.1%), and suicide (11.1%) among medical students is high [13, 14]. In Uganda, recent studies reported that one in five medical students are depressed, 54.5% of the students experience burnout, and 57.4% of the students in medical school are stressed [15–17]. Following the COVID-19 pandemic, the prevalence of mental illness among students increased with over four-fifths of the students having severe symptoms [17]. Students also reported high suicidal behaviours (i.e., suicidal ideations, attempts, and plans) [18, 19]. We hypothesized that the high levels of mental health problems are due to low levels of knowledge, poor attitude, and perception towards mental health care and mental health in general, which make students seek care late or avoid mental health services. For the present study, perceptions are one’s interpretation of a mental health challenges, while attitude is how one acts and behaves towards someone with mental health challenge [20].

Studies in some parts of sub-Saharan Africa have found that medical students and health workers have lower levels of knowledge, poor perception, and attitude towards mental illness [21, 22]. This not only predisposes them to mental illness but also hinders the utilisation of mental health services available and the recognition of mental illness in the people they serve [8, 23–27]. The poor perceptions and attitudes on the other hand may bring about the stigmatisation of patients who may shun away from treatment as a result. There is a paucity of information on the knowledge, attitude, and perceptions of medical students on mental health in Uganda. Given the high prevalence of mental illness amongst medical students in the country [15, 16, 28, 29] and the scarcity of human resources in the mental healthcare field [4], it is important for medical students to be knowledgeable and have the right attitude and perceptions about the subject. Our study sought to determine the student’s knowledge, attitudes and perspectives towards mental health.

## Methods

### Study design, area, and population

This was a cross-sectional quantitative study that was done between September and October 2021 and that conveniently recruited undergraduate students pursuing Bachelor of Medicine and Bachelor of Surgery at Mbarara University of science and technology, 18 years and above possessing a valid university ID.and were registered for the 2020/2021 academic year. MUST is a public university in southwestern Uganda and it is the second largest, oldest, and among the 12 universities training medical doctors in the country. As of January 2021, the MUST medical school curriculum runs for 5 years with students spending two years in pre-clinical training and three in clinical training at the Mbarara Regional Referral and Teaching Hospital. The medical students are exposed to psychiatry training in their fourth year.

### Sample size determination and sampling

The minimal sample size required to produce statistical power of 80% was calculated using Epi Info StatCalc for population surveys version 7.2.2.6. Using a population size of approximately 442 undergraduate university medical students as of 2021/2022, the expected prevalence of utilisation of mental health services at 50% since there was no readily available literature in a similar setting with an acceptable margin of error of 5% and design effect of 1.0, the minimum calculated sample size was 206. Convenience sampling was used to get study participants.

### Study procedure

A *Google Form* with the study tool was designed with an informed consent form on the first page clearly explaining the details of the study. The link to the form was circulated in the study population using the *WhatsApp* class groups and emailing platforms. Upon access to the form, the participants were required to give informed consent and thereafter asked to fill a semi-structured questionnaire with socio-demographic characteristics, other possible associated factors including utilisation of mental health services, tools for assessing knowledge, attitude, perception, and stigma towards mental illness. Students who didn’t give informed consent were taken straight to the end of the form with the option ‘submit’ without going through the questionnaire.

### Study variables

#### Independent variables

Sociodemographic factors like age, gender, year of study, marital status, history of mental health, family history of mental health, history of a classmate with mental illness, history of use of mental health services and source of mental health information (formal education, social media, books, seminar, environment, counsellors, trained personnel, leaders as well as podcasts and videos.)

#### Dependent variables

Our questionnaire was adopted from previous similar studies which explored knowledge, attitude, perceptions, and awareness of metal health problems among students [30]. It was then edited according to our particular population following a pilot study done among some medical students. Our dependent variables were knowledge, attitude and perceptions. Knowledge was assessed using “yes” or “no” with yes answers scoring 1 and no answers scoring 0 and Cronbach alpha score 0.5662. The scores ranged from a minimum of 0 to a maximum of 20. The participants’ attitudes toward people with mental disorders were assessed using 33 statements with a five-point Likert scale from 1(strongly disagree) and 5 (strongly agree) and Cronbach alpha score 0.8730. The scores were calculated for each respondent, with the total scores ranging from 33 to 165 points. Perceptions toward people with mental disorders were assessed using 16 statements with a Likert scale from 1 (strongly disagree) to 5 (strongly agree) and Cronbach alpha score 0.8169. Each respondent’s scores were calculated, resulting in total perception scores ranging from 16 to 80 points.

### Ethics

The study received ethical approval from the Research Ethics Committee of MUST (MUST-2021-99). Permission to collect data from participants was granted by the dean of students at MUST. All participants provided voluntarily gave informed consent to be enrolled into the study enrolment.

### Statistical analysis

Fully completed questionnaires were downloaded from *Google Forms* and exported into *Microsoft Excel 2016* for cleaning. The cleaned data was then exported to STATA version 17 for analysis STATA 17.0 (Stata Corp, College Station, Texas, USA). Numerical data were summarised as means and standard deviations for normally distributed variables or median and interquartile ranges for non-parametric variables. Categorical data were summarised as frequencies and proportions. Attitude, perceptions, and knowledge were classified based on the median score i.e., positive attitude if the respondents’ scores were ≥ 90, and negative if their scores were < 90 (median = 90). Since the median score for perceptions was 46, scores ≥ 46 corresponded to a positive perception, and scores < 46 entailed a negative perception. The participants were classified as having high knowledge > 16, low knowledge < 16, or moderate knowledge = 16. Based on the total scores we performed inferential statistics to determine the statistical difference between the level of knowledge, attitudes and perceptions. Initially, unadjusted bivariate linear regression analyses were performed to assess the association between the dependent and independent variables. Variables with a *p*-value of < 0.05 were transferred to multilevel linear regression to control for the effect of confounders and estimate the independent predictors of knowledge, attitude and perceptions towards mental health. A regression model was made by a backward stepwise logistic regression procedure. Odds ratios with 95% confidence intervals were employed to determine the strength of association between the outcome and explanatory variables. All tests were two-tailed, and a cut-off point was set at a p-value < 0.05.

## Results

### Student characteristics

Overall, 259 students participated in the study, with a response rate of 81.46%. Participants were aged between 19 and 37 years with a mean age of 23.12(± 2.45). The majority of the participants were male 63.59%, and the second years had the highest number of participants 22.27% (n = 47). About 41.71% of the participants reported having had a history of mental illness, and 62.09% reported having had a classmate with mental illness. However, only 13.27% of the participants reported using mental health services offered by the university. The majority of the participants obtained information on mental health from social media 68.25%. (Table 1)

**Table 1:** Distribution of study variables with knowledge, attitude and perceptions of students towards mental health

Categorical variable	n (%)	Knowledge (µ ± SD) 15.84 ± 2.29	X^2^ /t (***p*** value)	Attitude (µ ± SD) 91.49 ± 18.65	X^2^ /t (***p*** value)	Perceptions (µ ± SD) 45.16 ± 10.80	X^2^ /t (***p*** value)
**Age**	23.12 ± 2.45	r^2^ = 0.04	0.561	r^2^ = 0.18	**0.009**	r^2^ = 0.12	0.092
**Gender**							
Female	77(36.49)	16.05 ± 2.49	1.00(0.318)	88.01 ± 15.31	**-2.06(0.040)**	44.16 ± 10.03	-1.02 (0.309)
Male	134(63.59)	15.84 ± 2.17		93.49 ± 20.10		45.73 ± 11.23	
**Marital status**							
Cohabiting	12(5.69)	15.58 ± 4.06	0.23(0.874)	99.5 ± 24.38	1.50(0.215)	48.33 ± 12.73	1.24 (0.296)
Divorced	1(0.47)	17 ± 0		71 ± 0		55 ± 0	
Married	5(2.37)	16.4 ± 1.67		99.2 ± 12.93		51.2 ± 7.79	
Single	193(91.47)	15.84 ± 2.17		90.90 ± 18.29		44.75 ± 10.70	
**History of mental illness**							
Yes	88(41.71)	15.89 ± 2.18	-0.29(0.773)	88.28 ± 16.28	**2.13(0.034)**	42.15 ± 10.22	**3.50(0.001)**
No	123(58.29)	15.80 ± 2.38		93.78 ± 19.91		47.30 ± 10.71	
**Family history of mental illness**							
No	167(79.15)	15.99 ± 2.25	1.87(0.063)	91.51 ± 19.56	0.03(0.97)	45.44 ± 10.98	0.75(0.454)
Yes	44(20.85)	15.27 ± 2.41		91.41 ± 14.85		44.07 ± 10.120	
**History of classmate with mental illness**							
No	80(37.91)	15.56 ± 2.10	-1.58(0.115)	90.54 ± 19.75	-0.58(0.564)	44.31 ± 11.25	-0.89(0.376)
Yes	131(62.09)	16.04 ± 2.39		92.07 ± 17.98		45.68 ± 10.51	
**History of use of mental health services**							
No	183(86.73)	15.89 ± 2.24	0.67(0.501)	90.97 ± 19.26	-1.03(0.306)	45.15 ± 11.15	-0.03(0.976)
Yes	28(13.27)	15.57 ± 2.64		94.85 ± 13.74		45.21 ± 8.24	
**Year of study**							
1	41(19.43)	15.02 ± 2.45	**3.39(0.010)**	92.09 ± 16.26	**3.99 (0.004)**	43.73 ± 11.60	2.39(0.523)
2	47(22.27)	15.74 ± 2.26		86.68 ± 17.15		43.55 ± 9.72	
3	42(19.91)	16.02 ± 1.96		85.57 ± 18.19		43.07 ± 10.13	
4	43(20.38)	16.76 ± 1.67		95.63 ± 15.95		46.88 ± 10.38	
5	38(18.01)	15.60 ± 2.78		98.63 ± 22.90		49.02 ± 11.54	
**Sources of knowledge**							
**Formal education**							
No	81(38.39)	15.38 ± 2.37	**-2.33(0.02)**	88.75 ± 18.10	-1.69(0.092)	43.91 ± 9.72	-1.32(0.188)
Yes	130(61.61)	16.13 ± 2.20		93.19 ± 18.84		45.93 ± 11.38	
**Social media**							
No	67(31.75)	16 ± 2.46	0.68(0.50)	93.31 ± 17.38	0.97(0.333)	46.06 ± 10.53	0.83(0.408)
Yes	144(68.25)	15.77 ± 2.21		90.63 ± 19.20		44.74 ± 10.92	
**Books**							
No	112(53.08)	15.89 ± 2.46	0.33(0.74)	89.33 ± 15.75	-1.80(0.074)	44.22 ± 10.96	-1.34(0.182)
Yes	99(46.92)	15.78 ± 2.10		93.92 ± 21.27		46.21 ± 10.56	
**Seminars**							
No	158(74.88)	15.96 ± 2.29	1.23(0.22)	90.86 ± 18.02	-0.84(0.400)	45.32 ± 10.93	0.39(0.700)
Yes	53(25.12)	15.51 ± 2.30		93.35 ± 20.45		44.66 ± 10.46	
**Environment**							
No	141(66.82)	15.91 ± 2.11	0.64(0.52)	91.18 ± 17.45	-0.34(0.732)	45.58 ± 10.42	0.81(0.418)
Yes	70(33.18)	15.7 ± 2.63		92.11 ± 20.97		44.3 ± 11.53	
**Counselors**							
No	210(99.53)	15.86 ± 2.28	n/a	91.55 ± 18.67	n/a	45.17 ± 10.81	n/a
Yes	1(0.47)	12 ± 0		78 ± 0		42 ± 0	
**Trained personnel**							
No	210(99.53)	15.84 ± 2.29	n/a	91.48 ± 18.68	n/a	45.15 ± 10.82	n/a
Yes	1(0.47)	16 ± 0		92 ± 0		45 ± 0	
**Leaders**							
No	210(99.53)	15.84 ± 2.30	n/a	91.47 ± 18.68	n/a	45.20 ± 10.81	n/a
Yes	1(0.47)	17 ± 0		95 ± 0		37 ± 0	
**Podcasts and videos**							
No	210(99.53)	15.84 ± 2.30	n/a	91.55 ± 18.67	n/a	45.15 ± 10.82	n/a
Yes	1(0.47)	16 ± 0		78 ± 0		45 ± 0	

#### Knowledge of students about mental health

The average level of knowledge score of the study participants about mental health was 15.84 ± 2.29, with a minimum score of 4 and a maximum score of 19. About 77.72% (n = 164) had high knowledge on mental health, 10.43% (n = 22) had moderate knowledge, and 11.85% (n = 25) had little knowledge. Students whose source of knowledge about mental health was formal education, had more knowledge compared to those who did not obtain knowledge from formal education (16.13 ± 2.20 vs. 15.38 ± 2.37, t=-2.33, p = 0.02). (Table 1**).**

#### Attitudes of students towards mental health

Attitude was evaluated as how students related to issues concerning mental health. The mean score for attitude towards mental health was 91.49 ± 18.65, range of 42 to 150. More than half of the participants had a negative attitude towards mental health 50.71% (n = 107) with 49.29% (n = 104) having a positive attitude. Male medical students significantly had better attitudes towards mental health than females (93.49 ± 20.10 vs. 88.01 ± 15.31, t=-2.06, p = 0.040). (Table 1).

#### Perceptions of students towards mental health

Perception was assessed based on what students considered were truths about mental health and what it actually is. The average score on the assessment of perceptions of the students was 45.16 ± 10.80. range of 18 to 70. A total of 99 students (46.92%) had a positive perception of mental health. About 53.08% (n = 112) of the students had negative perceptions of mental health while 46.92% (n = 99) had positive perceptions of mental health. Students with no history of mental illness had better perceptions of mental illness than those with mental illness (47.30 ± 10.71 vs. 42.15 ± 10.22, t = 3.50, p = 0.001) (Table 1).

## Factors associated with knowledge, attitudes, and perceptions of participants towards mental illness

### Knowledge of students about mental illness

Following unadjusted linear regression analysis (Table 2), being in year 3, year 4, and having formal education as a source of knowledge for mental health were found to be associated with increased levels of knowledge. At multilevel linear regression, being in year 4 significantly increased the level of knowledge (Adjusted coefficient 1.50 [95% confidence interval = 0.46–2.54, *p* = 0.005). (Table 3).

**Table 2:** Unadjusted linear regression analysis displaying factors associated with knowledge, attitudes and perceptions of participants towards mental illness

Variable	Knowledge		Attitude		Perceptions	
	**Coefficient (95% confidence interval)**	***p*** **-value**	**Coefficient (95% confidence interval)**	***p-*** **value**	**Coefficient (95% confidence interval)**	***p*** **- value**
**Age**	-0.02 (-0.15–0.11)	0.759	1.30 (0.28–2.33)	**0.013**	0.57 (-0.03–1.16)	0.062
**Gender**						
Female	Ref		Ref		Ref	
Male	-0.33 (-0.97–0.32)	0.318	5.47 (0.26–10.69)	**0.040**	1.28(-1.47–4.62)	0.309
**Marital status**						
Cohabiting	Ref		Ref		Ref	
Divorced	1.42 (-3.31–6.14)	0.556	-28.5 (-66.62–9.62)	0.142	6.67 (-15.25–28.78)	0.553
Married	0.82 (-1.60–3.23)	0.506	-0.03 (-19.79–19.19)	0.976)	2.87 (-8.44–14.17)	0.618
Single	0.26 (-1.10-1.61)	0.709	-8.60 (-19.50–2.29)	0.121	-3.58 (-9.90–2.73)	
**History of mental health**						
No	Ref		Ref		Ref	
Yes	0.93 (-0.54–0.73)	0.773	-5.50 (-10.59 – -0.41)	**0.034**	-5.141 (-8.04 – -2.25)	**0.001**
**Family history of mental health**						
No	Ref		Ref		Ref	
Yes	-0.72 (-1.48–0.04)	0.063	-0.10 (-6.34–6.14)	0.975	-1.37 (-4.98–2.24)	0.454
**History of classmate with mental illness**						
No	Ref		Ref		Ref	
Yes	0.51 (-0.13–1.15)	0.115	1.53 (-3.69–6.76)	0.564	1.35 (-1.66–4.38)	0.376
**History of use of mental health services**						
No	Ref		Ref		Ref	
Yes	-0.31 (-1.23–0.60)	0.501	3.88 (-3.57–11.34)	0.306	0.06(-4.26–4.39)	0.976
**Year of study**						
1	Ref		Ref		Ref	
2	0.72 (-0.22–1.66)	0.134	-5.42 (-13.06–2.22)	0.164	-0.18 (-4.67–4.31)	0.938
3	0.99 (0.03–1.96)	**0.044**	-6.53 (-14.38–1.32)	0.103	-0.66 (-5.27–3.95)	0.778
4	1.74 (0.78–2.70)	**< 0.001**	3.53 (-4.27–11.33)	0.374	3.15 (-1.43–7.74)	0.177
5	0.58 (-0.41–1.58)	0.251	6.53 (-1.51–14.59)	0.111	5.29 (0.56–10.02)	**0.028**
**Sources of knowledge**						
**Formal education**						
No	Ref		Ref		Ref	
Yes	0.74 (0.11–1.38)	**0.021**	4.44 (-0.74–9.62)	0.093	2.02 (-0.99–5.02)	0.187
**Social media**						
No	Ref		Ref		Ref	
Yes	-0.23 (-0.90–0.44)	0.500	-2.67 (-8.11–2.76)	0.333	-1.32 (-4.47–1.83)	0.408
**Books**						
No	Ref		Ref		Ref	
Yes	-0.10 (-0.73–0.52)	0.741	4.60 (-0.44–9.64)	0.074	1.99 (-0.94–4.92)	0.182
**Seminars**						
No	Ref		Ref		Ref	
Yes	-0.45 (-1.16–0.27)	0.221	2.50 (-3.34–8.33)	0.400	-0.66 (-4.05–2.72)	0.700
**Environment**						
No	Ref		Ref		Ref	
Yes	-0.21(-0.88–0.45)	0.523	0.94 (-4.44–6.32)	0.732	-1.28 (-4.40–1.83)	0.418
**Counsellors**						
No	Ref		Ref		Ref	
Yes	-3.86 (-8.37–0.65)	0.093	-13.55 (-50.44–23.33)	0.470	-3.17 (-24.55–18.20)	0.770
**Trained personnel**						
No	Ref		Ref		Ref	
Yes	0.16 (-4.38-4.69)	0.946	0.51 (-36.41–37.44)	0.978	-0.16 (-21.54–21.23)	0.988
**Leaders**						
No	Ref		Ref		Ref	
Yes	1.16 (-3.38–5.70)	0.614	3.53 (-33.40–40.45)	0.851	-8.20 (-29.55–13.15)	0.450
**Podcasts and videos**						
No	Ref		Ref		Ref	
Yes	0.16 (-4.38–4.69)	0.946	-13.55 (-50.44–23.33)	0.470	-0.16 (-21.54–21.23)	0.988

**Table 3:** Multilevel linear regression analysis displaying factors associated with knowledge, attitudes, and perception of participants towards mental illness

Variable	Knowledge		Attitude		Perception	
	**Adjusted coefficient (95% confidence interval)**	**P valuer**	**Adjusted coefficient (95% confidence interval)**	**P valuer**	**Adjusted coefficient (95% confidence interval)**	**P valuer**
**Age**			2.66(-0.76–6.07)	0.128		
**Gender**						
Female			Ref			
Male			2.79 (-2.85–8.43)	0.332		
**History of mental health**						
No			Ref		Ref	
Yes			-2.72 (-8.33–2.86)	0.339	-4.23(-7.44 - -1.03)	**0.010**
**Year of study**						
1	Ref				Ref	
2	0.64 (-0.30–1.60)	0,281			0.70 (-3.69–5.09)	0.754
3	0.91 (-0.05–1.88)	0.063			-1.11 (-5.59–3.36)	0.634
4	1.5 0(0.46–2.54)	**0.005**			1.77 (-2.79–6.32)	0.447
5	0.42 (-0.59–1.43)	0.414			3.47 (-1.31–8.25)	0.155
**Formal education as source of knowledge**						
No	Ref					
Yes	0.38(-0.31–1.06)	0.281				

#### Attitudes of students towards mental health

Following unadjusted linear regression analysis (Table 2), older age and being male were associated with a better attitude towards mental health. However, having a history of mental illness was associated with lowering the attitude towards mental health. At multilevel analysis, none of the factors were associated with attitude towards mental health (Table 3).

#### Perceptions of students towards mental health

Following unadjusted linear regression analysis (Table 2), being in year 5 was associated with a positive perception of mental illness while having a history of mental illness worsened the perceptions. At multilevel analysis, a positive history of mental illness was found to be a predictor of worse perceptions of mental illness (Adjusted coefficient − 4.23 [95% confidence interval = -7.44 - -1.03], *p* = 0.010) (Table 3).

### Relationship between the outcome variables

There was a significant relationship between perception and attitude towards mental health (r^2^ = 0.62) and between knowledge and perception towards mental illness (r^2^ = 0.14). However, the relationship between attitude towards mental health and knowledge about mental illness was not significant .

## Discussion

This study found medical students in Uganda to have a high level of knowledge, whereas less than half had a positive attitude and good perceptions towards mental health. There was a significant positive relationship between attitude and perceptions towards mental illness. Being a fourth-year medical student was associated with a higher level of knowledge while having a positive history of mental illness was a predictor for worse, more negative perceptions of mental illness. In our study, higher scores of knowledges were associated with being a fourth-year medical student which coincides with the introduction of psychiatry in the curriculum at this level. This is similar to other studies that reported an increase in knowledge of medical students on mental health with age and year of study [30–33]. It is also found that students in third year onwards are exposed to information on mental health directly or indirectly especially as they begin to interface with the management of patients [31]. With this dynamic interaction being the major source of knowledge for the medical students, there is need for interventions to formally teach students about mental health right from year 1. This could ensure that students take care of their mental health throughout medical school and avoid mental illnesses. This study, as compared to a similar one among university students in Indonesia whose knowledge on mental health was found to be 50.23%, showed that a larger proportion as high as 77.72% was knowledgeable [30]. Lower statistics were also noted in Burkina Faso where they reported average knowledge [32]. The difference may be because the study in Indonesia also included non-medical students who are not taught about mental health, despite using similar questions in assessment of knowledge [30]. Our study also showed that majority of the students obtained information on mental health through social media (68.25%). This is mainly due to the fact that they get to read about various experiences of their peers and share freely among each other with subsequent support. A review of articles that evaluated the use of social networking sites like Facebook among adolescents and adults showed that these platforms were highly engaging, supportive and eliminative of some of the barriers young people faced in accessing mental health information [34] With the rising global trend with a key focus on improving of mental health, it was found that university students were interested in understanding the causes and consequences of poor mental health, and how to improve access to evidence base based support through student led research studies [35] This has paved way for the increase in the knowledge available for access by their peers on mental health.

Despite the study having only medical students – future advocates for mental health, over half (50.7%) had poor attitudes toward mental illness. The prevalence of poor attitudes was higher than that among university students in a study done in Indonesia (47.5%) [30]. Students in our study had a poorer attitude towards mental illness than many studies among medical students from India, for example, they were less than 45.5%, and neutral attitudes (82/150 on attitude towards psychiatry scale) in India [36, 37]. The participants ‘positive attitude was found to improve with an increase in age. This improvement in attitude could be attributed to their exposure to psychiatric education and rotation [36, 37]. Contrary to the findings of Puspitasari et al. (2020), male respondents in this study had a better attitude than their female counterparts [30]. This is in line with the findings of [38] Abdulbari et al. (2011) which suggested that men are more positive towards those with mental illness [38]. Furthermore, a history of having had a mental illness was associated with having a poor attitude which may be attributed to the high levels of stigma associated with mental illness experienced by the students’ attitudes.

The perception of mental illness was poorer compared to that among students from Indonesia [30]. Perception of mental illness is what one thinks about mental illness after analysing some concrete logical facts about it and it is not highly subjective. It is, therefore, no surprise that students with a history of mental illness were associated with negative perceptions of mental illness. In fact, a study showed that 51% of medical students who perceived having a mental health diagnosis were not willing to disclose this information due to fear of repercussions [39]. The completion of clerkship in psychiatry rotation among medical students did not improve the level of stigma toward people with mental illness in a study done among medical schools in Tunisia which showed that there is need to improve the curriculum to shape students’ responses better [40].

### Limitations

The findings in this study should be interpreted with the following limitations in mind. First, we used a cross-sectional study design making causality impossible. We recommend future researchers conduct follow-up studies to show how exposure to formal knowledge about mental illness in year 4 increases people’s knowledge, attitudes, and perceptions towards mental illness. Second, the study tool adopted was previously used in another study [30], but these tools have not been validated for use in medical students in our setting. This could have led to inaccurate estimations of the knowledge, attitude, and perceptions. Lastly, the sample comprised of students from one university, not representative of the whole population of medical students in Uganda.

## Conclusion

Generally, medical students had a high level of knowledge obtained from various sources including curriculum (introduced in year four), social networking sites, history of mental illness and research which helped to shape their attitudes and perceptions to a greater extent. It agrees with similar studies that there is need to introduce medical students early in their training to the existing mental health information in order to improve their response to recognition, management and accessibility of helpful services within and outside their universities for better health outcomes. The information present in this study can be used by policymakers and future researchers to design future studies and interventions to improve knowledge, perceptions, and attitudes, especially among students who have a history of mental illness. We recommend the mental health providers, educators, and policy makers at universities to aim at empathy educational strategies to improve knowledge, change attitudes, and perception for medical students towards mental illness.

## Electronic supplementary material

Below is the link to the electronic supplementary material.

Supplementary Material 1

Supplementary Material 2

Supplementary Material 3

## Data Availability

The data set used in this manuscript has been provided as a supplementary file.

## References

[1] World Health Organization (WHO). Depression and Other Common Mental Disorders Global Health Estimates. 2017.

[2] Charlson FJ, Diminic S, Lund C, Degenhardt L, Whiteford HA (2014). Mental and Substance Use Disorders in Sub-Saharan Africa: Predictions of Epidemiological Changes and Mental Health Workforce Requirements for the Next 40 Years. PLoS ONE.

[3] Whiteford HA, Degenhardt L, Rehm J, Baxter AJ, Ferrari AJ, Erskine HE (2013). Global burden of disease attributable to mental and substance use disorders: fi ndings from the Global Burden of Disease Study 2010. Lancet.

[4] Kaggwa MM, Rukundo GZ, Wakida EK, Maling S, Sserumaga BM, Atim LM (2022). Suicide and Suicide Attempts Among Patients Attending Primary Health Care Facilities in Uganda: A Medical Records Review. Risk Manag Healthc Policy.

[5] Kaggwa MM, Nkola R, Najjuka SM, Bongomin F, Ashaba S, Mamun MA (2021). Extrapyramidal Side Effects in a Patient with Alcohol Withdrawal Symptoms: A Reflection of Quality of the Mental Health Care System. Risk Manag Healthc Policy.

[6] Auerbach RP, Alonso J, Axinn WG, Cuijpers P, Ebert DD, Green JG (2016). Mental disorders among college students in the World Health Organization World Mental Health Surveys. Psychol Med.

[7] Nakku JEM, Rathod SD, Garman EC, Ssebunnya J, Kangere S, Silva M, De, et al. Evaluation of the impacts of a district – level mental health care plan on contact coverage, detection and individual outcomes in rural Uganda : a mixed methods approach. Int J Ment Health Syst. 2019;:1–13. 10.1186/s13033-019-0319-2 PMC6767634 31583013

[8] Iqbal MZ, Rathi R, Prajapati SK, Mavis SZQ, Pheng TS, Kee HW (2021). Knowledge, attitude, and practice about mental health challenges among healthcare students of a private university. J Pharm Bioallied Sci.

[9] Kaggwa MM, Muwanguzi M, Nduhuura E, Kajjimu J, Arinaitwe I, Kule M (2021). Suicide among Ugandan university students: evidence from media reports for 2010–2020. BJPsych Int.

[10] MacLean L, Booza J, Balon R (2016). The Impact of Medical School on Student Mental Health. Acad Psychiatry.

[11] Jacob R, Li TY, Martin Z, Burren A, Watson P, Kant R (2020). Taking care of our future doctors: A service evaluation of a medical student mental health service. BMC Med Educ.

[12] Khanna P, Roberts C, Lane AS (2021). Designing health professional education curricula using systems thinking perspectives. BMC Med Educ.

[13] Rotenstein LS, Ramos MA, Torre M, Bradley Segal J, Peluso MJ, Guille C (2016). Prevalence of depression, depressive symptoms, and suicidal ideation among medical students a systematic review and meta-analysis. JAMA - J Am Med Assoc.

[14] Quek TTC, Tam WWS, Tran BX, Zhang M, Zhang Z, Ho CSH, et al. The global prevalence of anxiety among medical students: A meta-analysis. Int J Environ Res Public Health. 2019;16. 10.3390/ijerph16152735 PMC6696211 31370266

[15] Olum R, Nakwagala FN, Odokonyero R (2020). Prevalence and factors associated with depression among medical students at Makerere university, Uganda. Adv Med Educ Pract.

[16] Kajjimu J, Kaggwa MM, Bongomin F (2021). Burnout and associated factors among medical students in a public university in Uganda: A cross-sectional study. Adv Med Educ Pract.

[17] Najjuka SM, Checkwech G, Olum R, Ashaba S, Kaggwa MM (2021). Depression, anxiety, and stress among Ugandan university students during the COVID-19 lockdown: An online survey. Afr Health Sci.

[18] Kaggwa MM, Arinaitwe I, Muwanguzi M, Nduhuura E, Kajjimu J, Kule M, et al. Suicidal behaviours among Ugandan university students: a cross-sectional study. BMC Psychiatry 2022 221. 2022;22:1–13. 10.1186/s12888-022-03858-7 PMC8972906 35365105

[19] Kaggwa MM, Arinaitwe I, Nduhuura E, Muwanguzi M, Kajjimu J, Kule M (2022). Prevalence and Factors Associated With Depression and Suicidal Ideation During the COVID-19 Pandemic Among University Students in Uganda: A Cross-Sectional Study. Front Psychiatry.

[20] Sanjaya E, Sukarmin Y. The Differences in Audiences’ Attitude, Perception, and Loyalty based on Gender towards Volleyball League Matches in Yogyakarta, Indonesia. 2020;:540–6.

[21] Chukwujekwu CD (2018). Knowledge and Attitude about Mental Illness of Students in a University in Southern Nigeria. J Biosci Med.

[22] Minty Y, Moosa MYH, Jeenah FY. Mental illness attitudes and knowledge in non-specialist medical doctors working in state and private sectors. S Afr J Psychiatr. 2021;27. 10.4102/sajpsychiatry.v27i0.1592 PMC8182464 34192080

[23] Pinto IC, Bernardo M, Sousa S, Curral R (2020). Evaluation of mental health stigma on medical education: an observational study with Portuguese medical students. Porto Biomed J.

[24] Tjia J, Givens JL, Shea JA (2005). Factors associated with undertreatment of medical student depression. J Am Coll Heal.

[25] Chew-Graham CA, Rogers A, Yassin N (2003). “I wouldn’t want it on my CV or their records”: Medical students’ experiences of help-seeking for mental health problems. Med Educ.

[26] Vidourek RA, King KA, Nabors LA, Merianos AL (2014). Students’ benefits and barriers to mental health help-seeking. Heal Psychol Behav Med.

[27] Okpalauwaekwe U, Mela M, Oji C, Udokaokpala. Knowledge of and Attitude to Mental Illnesses in Nigeria: A Scoping Review. In: Integrative Journal of Global Health. 2017. p.5.

[28] Bacchi S, Licinio J (2015). Qualitative Literature Review of the Prevalence of Depression in Medical Students Compared to Students in Non-medical Degrees. Acad Psychiatry.

[29] Amanya SB, Nakitende J, Ngabirano TD (2018). A cross-sectional study of stress and its sources among health professional students at Makerere University, Uganda. Nurs Open.

[30] Puspitasari IM, Garnisa IT, Sinuraya RK, Witriani W (2020). Perceptions, knowledge, and attitude toward mental health disorders and their treatment among students in an Indonesian University. Psychol Res Behav Manag.

[31] Aruna G, Mittal S, Yadiyal MB, Acharya C, Acharya S, Uppulari C (2016). Perception, knowledge, and attitude toward mental disorders and psychiatry among medical undergraduates in Karnataka: A cross-sectional study. Indian J Psychiatry.

[32] Sawadogo KCC, Lameyre V, Gerard D, Bruand PE, Preux PM (2020). Knowledge, attitudes and practices in mental health of health professionals at the end of their curriculum in Burkina Faso: A pilot study. Nurs Open.

[33] Riffel T, Chen SP. Exploring the Knowledge, Attitudes, and Behavioural Responses of Healthcare Students towards Mental Illnesses—A Qualitative Study. Int J Environ Res Public Heal 2020, Vol17, Page 25. 2019;17:25. 10.3390/ijerph17010025 PMC6981946 31861420

[34] Ridout B, Campbell A. The use of social networking sites in mental health interventions for young people: Systematic review. J Med Internet Res. 2018;20. 10.2196/12244 PMC6315265 30563811

[35] Sampson K, Priestley M, Dodd AL, Broglia E, Wykes T, Robotham D (2022). Key questions: research priorities for student mental health. BJPsych Open.

[36] Poreddi V, Thimmaiah R, Math SB (2015). Attitudes toward people with mental illness among medical students. J Neurosci Rural Pract.

[37] Poreddi V, Thimmaiah R, BadaMath S (2017). Medical and nursing students’ attitudes toward mental illness: An Indian perspective. Investig y Educ en Enferm.

[38] Abdulbari B, Suhaila G (2011). Gender differences in the knowledge, attitude and practice towards mental health illness in a rapidly developing Arab society. Int J Soc Psychiatry.

[39] Fletcher I, Castle M, Scarpa A, Myers O, Lawrence E. An exploration of medical student attitudes towards disclosure of mental illness. Med Educ Online. 2020;25. 10.1080/10872981.2020.1727713 PMC7034470 32054420

[40] Brahmi L, Amamou B, Ben Haouala A, Mhalla A, Gaha L (2022). Attitudes toward mental illness among medical students and impact of temperament. Int J Soc Psychiatry.

