# Supplementary material for: Knowledge, attitude and perceptions of medical students towards mental health in a university in Uganda
Source: BMC Med Educ. 2022 Oct 20;22:730. doi: 10.1186/s12909-022-03774-0 (PMC9584261; doi:10.1186/s12909-022-03774-0)
Supplement: Supplementary file 2 — Supplementary Material 2 [file 12909_2022_3774_MOESM2_ESM.pdf]

1.0. Student questionnaire

Question	Response	
Age		
Gender	- Male<br>- Female	
Marital status	- Single<br>- Cohabiting<br>- Married<br>- Separated<br>- Divorced	
Religion	- Christian<br>- Moslem	
Year of study	- 1<br>- 2<br>- 3<br>- 4<br>- 5	
Positive history of mental illness	- Yes<br>- No	
History of a family member with mental illness	- Yes<br>- No	
History of a friend or a classmate with mental illness	- Yes<br>- No	
History of use of mental health services	- Yes<br>- No	
Source of information about mental health	- Social media<br>- Formal education<br>- Environment<br>- Books<br>- Seminars	
Knowledge about mental health services		
	Yes	No
Exercise can help maintain mental health		
Mental disorders are caused by wrong way of thinking		
Many people have psychiatric problems, but they do not realize them		
External stress factors are the causes of all types of mental health disorders		
The components of mental health include normal intelligence, stable moods, positive attitudes,		

interpersonal relationships, and quality adaptability		
The majority of mental disorders cannot be cured		
Psychological or psychiatric services should be sought if one suspects the presence of psychological problems or mental disorders		
Psychological problems can occur at almost all ages		
Mental disorders and psychological problems cannot be prevented		
In severe mental disorders (eg, schizophrenia), treatment is only given within a certain period of time and should not be given for a long period of time		
The main symptom of schizophrenia is hallucination		
Individuals who have a family history of mental disorders have a higher risk of experiencing psychological problems and mental disorders		
Psychological problems in adolescents do not affect academic grades		
Middle-aged or elderly individuals rarely have psychological problems and mental disorders		
Individuals with bad temperament are more likely to have psychiatric problems		
Feelings of sadness and depression are the same		
The treatment of people with mental health disorders is enough by giving antidepressants		
The treatment of people with mental health disorders needs supportive psychological therapy		
Mental health medications do not provide considerable adverse effects		
Sertraline is one of the antidepressants		
**Students' Attitudes**		

Question	Strongly agree	Agree	Neither agree or disagree	Disagree	Strongly disagree
People with mental illnesses deserve respect					
We must help people with mental illnesses for them to be better					
A mockery of mental disorders is painful					
Learning about mental illnesses is crucial.					
Avoiding people with mental illnesses is a good idea.					
I feel comfortable when encountering people with mental illnesses.					
People with mental illnesses can help others.					
I am scared when being approached by people with mental illnesses.					
When I have a mental health disorder, I most likely do not tell my friends.					
If any of my friends suffer from mental illnesses, then I would advise them not to tell anyone					
Caring for people with mental illnesses in hospitals makes the community feel safer.					
Only people who are weak and overly sensitive let themselves be affected by mental illnesses					
It would be a shame if I had a mental illness.					
Students with mental illnesses should not be in regular classes.					
I have a little in common with people suffering from mental health disorders.					
Students with mental illnesses need a special curriculum in learning.					
Someone with mental illnesses can be a good friend.					
I just learn about psychiatry because it is in the exam and would not bother reading additional material on it					

People with severe mental illness can never recover enough to have a good quality of life					
Psychiatry is as scientific as other fields of medicine					
If I had a mental illness, I would never admit this to any of my friends because I would fear being treated differently					
People with mental illness are dangerous more often than usual					
Psychiatrists know more about the lives of people they treat with mental illness, compared with their carers (family members or friends of people with mental illness)					
Being a psychiatrist is not like being a real doctor					
If a consultant psychiatrist instructed me to treat people with mental illness in a derogatory manner, I would not follow the consultant's instructions					
I feel as comfortable talking to a person with mental illness as I do a person with physical illness					
It is important that any doctor supporting a person with mental illness assess the physical health of a person with mental illness					
The public does not need to be protected from people with mental illness					
If a person with mental illness complained of physical symptoms such as chest pain, it is usually part of their mental illness					
General practitioners should not be expected to complete a thorough assessment of people with psychiatric symptoms because they are referred to a psychiatrist					
I would not use the term "crazy", "nutter", "mad" etc to describe people with mental illness that I have seen on the ward to colleagues					

If my colleague told me they had a mental illness, I would still want to work with them					
If I had a mental illness I would never admit this to my colleagues for fear of being treated differently					
**Students' Perceptions**					
Response	Strongly agree	Agree	Neither agree or disagree	Disagree	Strongly disagree
People with mental health problems tend to be blamed for their conditions.					
One can tell whether an individual has a mental health disorder through his/her physical appearance.					
People who are mentally ill cannot make friends.					
People with mental illnesses can work.					
People with mental illnesses are commonly dangerous					
Anyone can suffer from a mental illness.					
People with mental illnesses are insane.					
Do you agree that substance misuse like alcohol or drug could result in mental illness?					
Do you agree that genetic inheritance could be the cause of mental illness?					
Do you agree head injury can be the cause of mental illness?					
Do you agree that physical illness (like diabetes or HIV/AIDS) can be the cause of mental illness					
Do you agree mental illness is treatable?					
Do you agree that stress in daily life can cause mental illness?					
Do you agree mental illness is contagious?					
Do you agree mental illness is a punishment from God?					
Do you agree the evil spirit can be the cause of mental illness?					
